# β-sitosterol induces reactive oxygen species-mediated apoptosis in human hepatocellular carcinoma cell line

**DOI:** 10.22038/AJP.2021.17746

**Published:** 2021

**Authors:** Mary J. Ditty, Devaraj Ezhilarasan

**Affiliations:** 1 *Department of Pharmacology, The Blue Lab, Molecular Medicine and Toxicology Division, Saveetha Dental College, Saveetha Institute of Medical and Technical Sciences, Chennai, Tamil Nadu, India*

**Keywords:** Liver cancer, β-sitosterol, Reactive oxygen species, Apoptosis, Caspase

## Abstract

**Objective:**

It is of interest to investigate the anti-proliferative effect of β-sitosterol (BS) on human hepatocellular carcinoma (HepG2) cell line.

**Materials and Methods:**

β-sitosterol treatments (0.6 and 1.2 mM/ml) were done in HepG2 and after 24 hr, cell viability was evaluated by MTT assay. Reactive oxygen species (ROS) accumulating potential of BS was assessed by dichloro-dihydro-fluorescein diacetate staining. Morphology related to apoptosis was investigated by acridine orange and ethidium bromide dual staining. Cytochrome c and caspase 3 expressions were evaluated by immunofluorescence and western blot analyses.

**Results:**

β-sitosterol induced cytotoxicity (p<0.001) and intracellular ROS in HepG2 cells in a dose-dependent manner. BS treatments accumulated induced intracellular ROS accumulation which led to membrane damage and mitochondrial toxicity. At the molecular level, BS treatments induced cytochrome c release from mitochondria and enhanced the protein expressions (p<0.05 vs 0.6 mM/ml and p<0.001 vs 1.2 mM/ml) of both caspase 3 and cleaved caspase 3.

**Conclusion:**

β-sitosterol induced ROS accumulation which plays a critical role in apoptosis via the intrinsic pathway in HepG2 cells. The present investigation paves the way for further *in vivo* studies.

## Introduction

Natural products such as phytochemical compounds from medicinal plants are one of the best sources of drugs and many chemotherapeutic drugs existing today are plant-derived compounds (Ezhilarasan, 2018 ▶; Rayan et al., 2017 ▶). Phytosterols are structurally similar to cholesterol and are specific phytochemicals found only in plants. However, phytosterols have an extra hydrocarbon chain at the C-24 position which differs from cholesterol (Zaloga, 2015 ▶). β-sitosterol (24a-ethylcholesterol), stigmasterol (D22, 24a-ethylcholesterol), and (campesterol (24a-methylcholesterol) (Bacchetti et al., 2011 ▶) are main plant sterols that have beneficial roles in a variety of chronic diseases including cardio vascular disorders (Jones and AbuMweis, 2009 ▶), diabetes (Misawa et al., 2012 ▶), and cancer (Suttiarporn et al., 2015 ▶). It has been reported that intake of phytosterols-rich diets can reduce the cancer risk by 20% (Ramprasath and Awad, 2015 ▶). Therefore, evaluation of anticancer potential of these phytosterols merits further study. β-sitosterol (BS) is a well-studied plant-derived sterol known for its beneficial effects against liver diseases (Devaraj et al., 2020 ▶). Several experimental studies have shown anticancer potentials of BS (Choi et al., 2003 ▶; Baskar et al., 2012 ▶; Sharmila and Sindhu, 2017 ▶). Particularly *in vitro*, the anti-proliferative effect of BS has been reported against human colon (HT116) (Choi et al., 2003 ▶), lung (A549) (Rajavel et al., 2018 ▶), gastric adenocarcinoma (Shin et al., 2018 ▶), prostate (PC-3) (Awad et al., 2001 ▶), and breast (MCF-7) cancer cell lines (Awad et al., 2008 ▶). Among phytosterols, BS has been shown to enhance the effectiveness of standard chemotherapeutic agents (Awad et al., 2008 ▶; Cao et al., 2019 ▶).

Hepatocellular carcinoma (HCC) ranks third in causing cancer-related mortality responsible for 600,000 annual deaths globally (Siegel et al., 2016 ▶; Jiang et al., 2017 ▶). Chronic hepatitis B and C virus infections, chronic alcohol consumption, consumption of aflatoxin-contaminated food, certain metabolic liver diseases, and cirrhosis are considered primary risk factors of HCC (Suh et al., 2018). Current cancer treatments include surgical removal and radiotherapy, followed by systemic chemotherapy used for maintenance treatment (Ezhilarasan, 2018 ▶; Gheena and Ezhilarasan, 2019 ▶). The major drawbacks of chemotherapy are recurrence, drug resistance, and severe off-target side effects that limit the use of chemotherapeutic drugs in cancer patients (Solai Prakash and Devaraj E, 2019). Despite significant therapeutic advancements, to date, HCC remains most aggressive cancer type responsible for significant mortality rate worldwide (Hartke et al., 2017 ▶; Shebi et al., 2018 ▶). On the other hand, in a recent systematic meta-analysis, low dietary phytosterol intake has been correlated with high cancer risk (Jiang et al., 2019 ▶). Phytosterols dietary intervention effectively control cancer progression and reduces its risk (Alvarez-Sala et al., 2019 ▶). Therefore, this study investigates the cytotoxic potentials of BS against human HCC (HepG2) cell line.

## Materials and Methods

**Chemicals**

The cell cultures reagents such as Dulbecco’s minimum essential low glucose medium (DMEM), dimethyl sulfoxide (DMSO), antibiotics (penicillin and streptomycin), fetal bovine serum (FBS), tryspin-EDTA, and 3-(4, 5-dimethylthiazol-2-yl)-2, 5-diphenyltetrazolium bromide (MTT) were purchased from GIBCO-BRL (Gaithersburg, MD). β-sitosterol was commercially procured from Sigma chemical (Chennai, India). 

**Cell culture and maintenance**

The HepG2 cells were obtained from The NCCS, Pune, India. The DMEM medium containing 10% of FBS, penicillin and streptomycin was used for cell culture. Cells were maintained in a standard culture condition at 37°C with 5% CO_2_. After reaching enough confluence, 0.25% trypsin-EDTA was added to detach cells and cells were seeded for the treatment. After maintaining couple of passages, cells were used for treatments. The experiments were done once cells reached 70-80% confluence. 

**MTT assay**

Cytotoxicity inducing potential of BS was done by MTT assay (Ponselvi Induja et al., 2018 ▶). After cell counting, 1×10^4^ cell suspension was added to a 96-well plate. After cells adherence for 24 hr, the existing medium was changed to 100 µl of medium with or without BS (0.2, 0.4, 0.6, 0.8 and 1 mM/ml) and left for 24 hr. BS was dissolved in 0.1% DMSO and hence, it was served as internal control. After 24 hr, cells were washed once, 50 μl (0.5 mg/ml in PBS) of MTT was added to wells and cells were incubated at 37°C for 4 hr in CO_2_ incubator. After incubation, purple formazan product was dissolved by adding DMSO and the intensity of formazan product was measured by spectrophotometer (Biotek, India). 

**Reactive oxygen species (ROS) level analysis by dichloro-dihydro-fluorescein diacetate (DCFH-DA) staining**

BS-induced ROS accumulation in HepG2 cells was observed by a non-fluorescent probe DCFH-DA (Gheena and Ezhilarasan, 2019 ▶). After treatment, cells were detached by trypsin treatment. After counting, 8×10^6^ cells/well was incubated with DCFH-DA (10 µM) at room temperature for 30 min. After incubation, the cells were viewed instantly under an inverted microscope (Nikon Instruments Inc., NY, USA). 

**Acridine orange/ethidium bromide (AO/EB)** **staining and fluorescent microscopy**

AO/EB staining was performed according to Lakshmi et al. (2017). HepG2 cells were seeded at a concentration of 1×10^4^ in 48-well plates. After treatments with 0.1% DMSO and BS for 24 hr, cells were collected and used for AO/EB staining. Then, 100 μl of AO/EB (1:1) dyes was added to 100 μl of cell suspension, mixed well and investigated instantaneously under a fluorescence microscope (Nikon, Ti series, Japan). Cells from each samples were counted in different fields and the population of apoptotic cells percentage was calculated.

**Immunofluorescence analysis of cytochrome c**

In a 12-well plate, 5x10^4^ cells were treated with different BS concentrations and incubated for 24 hr. After washing thrice with PBS, and the cells were fixed in 4% formaldehyde exactly for 10-15 min. Cells were rehydrated, blocked with 5% normal goat serum, permeabilized using 0.5% Tween 20 and then incubated with monoclonal cytochrome C primary antibody (ab13575), followed by probing with goat anti-mouse IgG (ab150115) secondary antibody for 2 hr. Then, images were captured using Nikon Eclipse Ti inverted fluorescence microscope (Nikon Instruments Inc., NY, USA). 

**Western blot analysis of caspase 3**

After the treatment, control and BS treated cells were washed with PBS and were collected in radioimmunoprecipitation assay buffer (Sigma Aldrich, USA) and the lysed cells were mixed with sodium dodecyl sulfate (SDS) containing loading buffer (Thermo Scientific, USA). Then, it was incubated at 95^o^C for 5 min. After protein quantification, 50 µg of each samples was subjected to SDS-polyacrylamide gel electrophoresis (10%) for 90 min. The gel was then transferred onto polyvinylidene difluoride membranes and electro-transferred membrane was blocked with skimmed milk powder for 2 hr at room temperature. The membrane was incubated with anti-cleaved (activated) caspase-3 antibodies (monoclonal, IgG1, 1:100, Cell signaling technology, #9669, USA) overnight at 4^o^C. After incubation, membranes were incubated with corresponding secondary antibodies conjugated with horseradish peroxidase (1:2000) for 2 hr at room temperature. The membranes were developed by Pierce enhanced chemiluminescence plus western blotting substrate (Thermo Scientific, USA).

**Statistical analysis**

Values are presented as mean±S.E.M. (n=3) and were subjected to one-way ANOVA and Dunnett’s multiple comparison test was done to determine the statistical differences among groups. A p<0.05 was considered significant (Graph Pad prism 7.0. CA, USA).

## Results

**Cytotoxic potential of β-sitosterol in HepG2 cells**

**Figure 1:**
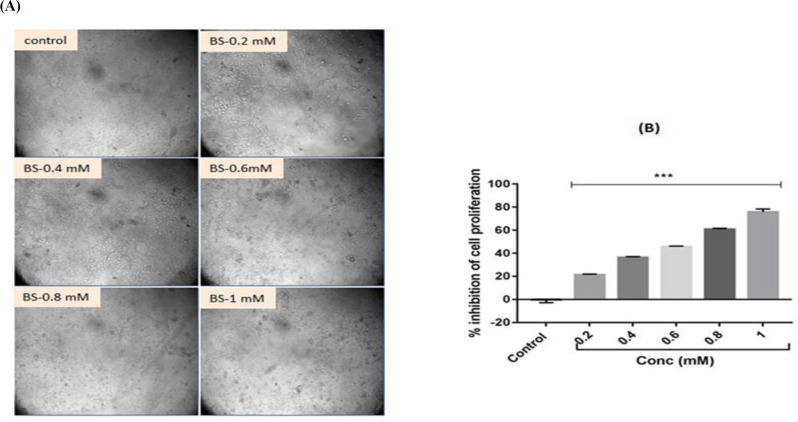
β-sitosterol (BS)-induced changes in the proliferation of HepG2 cells. A. Morphology of BS-treated HepG2 cells. B. Cytotoxicity analysis by MTT assay. n=3. ***p<0.001 vs control

To investigate the cytotoxic potential of BS in HepG2 cells, we performed MTT assay. Firstly, HepG2 cells were exposed to various concentrations of BS (0.2, 0.4, 0.8 and 1 mM/ml) for 24 hr and their cytotoxicity was assessed using MTT assay. The control and BS-treated cells morphology is depicted in Figure 1A. BS treatments significantly (p<0.001) induced dose-dependent cytotoxicity in HepG2 cells. The maximum cytotoxicity was evidenced at 1 mM/ml (Figure 1B). The IC_50_ value of BS for in HepG2 cells was 0.6 mM/ml at 24 hr. Therefore, further studies were carried out at concentrations of 0.6 and 1.2 mM/ml.

**β-sitosterol treatments induced intracellular ROS accumulation in HepG2 cells**

**Figure 2:**
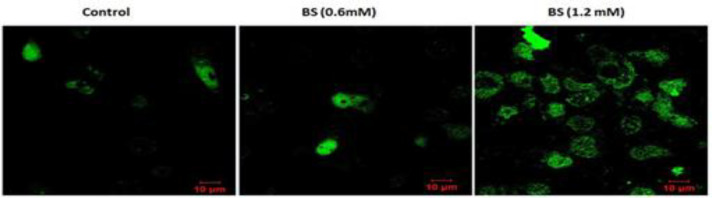
Reactive oxygen species inducing potentials of β-sitosterol (BS) in HepG2 cells as assessed by 2', 7'-dichlorodihydrofluorescein diacetate staining

Further, to find out whether BS-induced cytotoxicity was due to the accumulation intracellular ROS, we analyzed ROS by DCFH-DA assay. β-sitosterol at two different concentrations (0.6 and 1.2 mM/ml) for 24 hr, induced ROS expression. The ROS expression was not prominent at low concentration of BS treatment (*i.e.* 0.6 mM/ml) however, at high concentration, BS treatment prominently increased ROS expression suggesting that a high concentration of BS is required to induce ROS in HepG2 cells (Figure 2).

**β-sitosterol treatments induced apoptosis-related morphological changes**

**Figure 3:**
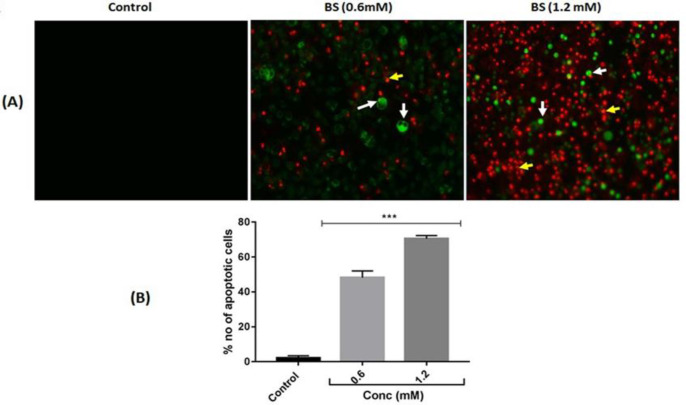
(A) Morphological analysis of apoptosis by acridine orange/ethidium bromide dual staining. (B). Quantification of early and late apoptotic cells. n=3. ***p<0.001 vs control

β-sitosterol treated HepG2 cells were exposed to AO/EB dual staining. The dye AO enters the nucleus and stains live cells as intense green color. While EB penetrates the nuclei of dead or late apoptotic cells due to membrane damage and stains as red color. In this study, early apoptotic cell appeared as greenish yellow colored nuclei with condensed or fragmented chromatin and late apoptotic cells nuclei appeared as red color with highly condensed or fragmented chromatin (Figure 3A). BS 

 treatments dose-dependently (p<0.001) increased the number of apoptotic cells as compared to control (Figure 3B).

**β-sitosterol-induced cytochrome c dislocation**

**Figure 4:**
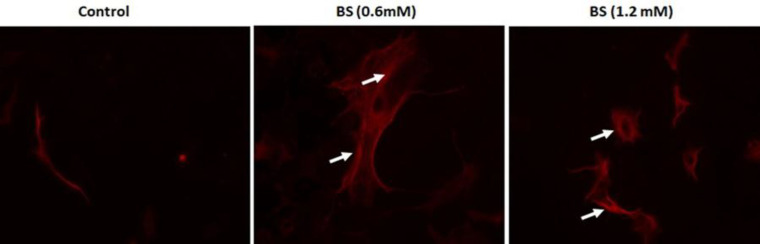
Immunofluorescence analysis of cytochrome c dislocation in control and β-sitosterol (BS)-treated HepG2 cells

 β-sitosterol-treated HepG2 cells were analyzed for cytochrome c expression as its

cytosolic expression plays a significant role in the intrinsic mitochondrial pathway. Cytochrome c was prominently expressed in BS-treated cells as compared to control cells. The maximum expression was noticed for 1.2 mM/ml of BS (Figure 4).

**Figure 5:**
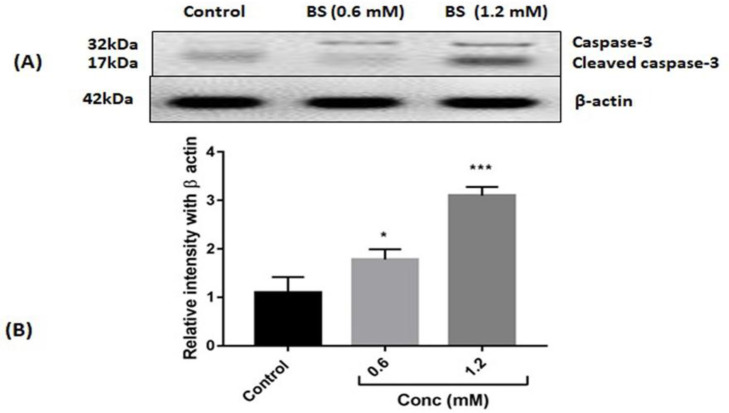
(A) Western blot expression of caspase 3 and its cleaved fraction. (B). Quantification of caspase protein expression by densitometry analysis. n=3. ***p<0.001 vs control

**β-sitosterol-induced caspase 3 and cleaved caspase expression**

Further to confirm the downstream target of cytochrome c, we analyzed caspase 3 expressions in BS-treated HepG2 cells. BS treated cells were significantly expressed cleaved caspase-3 and caspase-3. There was a prominent expression of both caspase 3 and cleaved caspase 3 in HepG2 cells treated with the high concentration of BS as compared to control (Figure 5A). Quantitative analysis by densitometry confirmed a significant increase of caspase expression p<0.05 vs 0.6 mM/ml and p<0.001 vs 1.2 mM/ml upon BS treatment in HepG2 cells (Figure 5B).

## Discussion

β-sitosterol and its oxy-derivatives have been reported to offer protection against cancer via induction of apoptosis, cytotoxicity, and cell cycle arrest and inhibition of adhesion metastasis, angiogenesis, and cell invasion (Ramprasath and Awad, 2015 ▶; Baskar et al., 2012 ▶; Li et al., 2016 ▶; Raj et al., 2020). In adjuvant therapy, the efficacy of gemcitabine, a standard chemotherapeutic agent was evidently increased when it was combined with BS (Cao et al., 2019 ▶). In previous studies, BS treatments inhibited the proliferation of lung, prostate, breast, gastric and colon cancer cell lines (Awad et al., 2001 ▶; Choi et al., 2003 ▶; Awad et al., 2008 ▶; Shin et al., 2018 ▶). Consistent with above studies, in this study, BS treatments dose-dependently caused cytotoxicity in HepG2 cells and these results suggest that regardless of cancer cell line, BS induces cytotoxicity.

Indeed, phytocompounds are capable of inducing ROS in cancer cells as compared to normal cells. Mitochondria act as both source and target for intracellular ROS. ROS can induce dissipation of mitochondrial membrane potential and cytochrome c release and it is one of the early events in apoptosis (Rajavel et al., 2018 ▶; Ezhilarasan et al., 2019 ▶; Gheena and Ezhilarasan, 2019 ▶; Rohit and Ezhilarasan, 2019 ▶). Therefore, we analyzed ROS expression to investigate whether oxidative stress was responsible for cytotoxicity induced by BS. The present study showed that BS could induce intracellular ROS level in HepG2 cells. In previous studies, BS treatments were shown to effectively induce ROS-mediated cytotoxicity in various cancer cell lines (Baskar et al., 2010 ▶; Kazłowska et al., 2013 ▶; Yang et al., 2013 ▶; Rajavel et al., 2018 ▶) and therefore, in light of these studies, the ROS inducing potential of BS could be hold accountable for its cytotoxic potential in HepG2 cells.

Phytocompounds selectively target cancer cells as they have a different stress response compared to normal cells. Phytocompounds have selective pro-oxidant effects on cancer cells (Chirumbolo et al., 2018 ▶). For instance, BS from *Grewia tiliaefolia *induced cytotoxicity only in human lung cancer cell lines (A549, MCF-7, PC3 and L-132) but not in normal human lung (L132) and peripheral blood mononuclear cells (Rajavel et al., 2017 ▶). Phytoderived cytotoxic drugs like paclitaxel, vincristine, vinblastine, and podophyllotoxin analogues are currently in clinical use against a variety of cancers (Hosseini and Ghorbani, 2015 ▶). The intracellular ROS accumulation is one of the direct causes of apoptosis induction and morphological damage induced by plant-derived chemotherapeutic drugs (Ezhilarasan et al., 2019 ▶; Rohit and Ezhilarasan, 2019 ▶) and therefore, we studied the BS-induced morphological changes related to apoptosis by AO/EB staining. In this study, BS-induced apoptotic cell death in HepG2 cells. AO/EB staining is a gold standard technique to detect cells undergoing apoptosis (Kasibhatla et al., 2006 ▶; Liu et al., 2015 ▶; Shebi et al., 2019 ▶). AO stain only enters live cells as they have intact cell membrane and emit intense green fluorescence in nuclei. EB enters cells which lost cytoplasmic membrane and intercalate with DNA thus, nuclei appear red. Early apoptotic cells with fragmented chromatin will have bright yellowish-green nuclei (Kasibhatla et al., 2006 ▶). Nuclear fragmented and chromatin condensed nuclei appear as bright green patches and late apoptotic cells show orange to red nuclei (Byczkowska et al., 2013 ▶). Consistent with previous studies, in this study, BS-treated cells showed red-colored nuclei which indicate cells with DNA damage which are in the late apoptotic phase. The appearance of intense red-colored nuclei after treatment with BS 1.2 mM/ml attributed to plasma membrane blebbing, and chromatin and nuclear condensation. These changes strongly indicated that BS-treated cells were undergoing apoptosis.

Apoptosis plays a crucial role in elimination of unwanted cells and maintaining homeostasis (Pfeffer and Singh, 2018 ▶; Li et al., 2019 ▶). Previous studies showed ROS-mediated apoptosis-inducing potential of phytochemicals in cancer cells (Ezhilarasan, 2018 ▶; Hong et al., 2019 ▶). Particularly, mitochondria are one of the main target of intracellular ROS and are also responsible for the generation of ROS intracellularly (Yang et al., 2016 ▶). The cytochrome c release from mitochondria is a key and early intracellular event during the mitochondrial apoptotic pathway (Jan and Chaudhry, 2019 ▶). In the cytosol, cytochrome c combines with Apaf–1 and procaspase-9 and form apoptosome. Then apoptosome subsequently activates caspase 9 and -3 signaling cascade to trigger apoptosis (Jan and Chaudhry, 2019 ▶). In the present investigation, BS caused an enhanced cytosolic expression of cytochrome c and caspase 3 and this could contribute to apoptosis due to the mitochondrial dysfunction and increased mitochondrial membrane permeability. 

In summary, our current results suggest that ROS production by BS plays a crucial role in apoptosis induction via intrinsic pathway in HepG2 cells. Therefore, these findings suggest that BS may be useful as an adjuvant drug in cancer chemotherapy for liver cancer patients. However, future *in vivo* studies and clinical trials are warranted to explore the detailed molecular mechanisms responsible for ROS generation and growth inhibitory effects of BS in liver cancer. 

## Conflicts of interest

The authors have declared that there is no conflict of interest.
